# Nilotinib interferes with cell cycle, ABC transporters and JAK-STAT signaling pathway in CD34+/lin- cells of patients with chronic phase chronic myeloid leukemia after 12 months of treatment

**DOI:** 10.1371/journal.pone.0218444

**Published:** 2019-07-18

**Authors:** Alessandra Trojani, Ester Pungolino, Alessandra Dal Molin, Milena Lodola, Giuseppe Rossi, Mariella D’Adda, Alessandra Perego, Chiara Elena, Mauro Turrini, Lorenza Borin, Cristina Bucelli, Simona Malato, Maria Cristina Carraro, Francesco Spina, Maria Luisa Latargia, Salvatore Artale, Pierangelo Spedini, Michela Anghilieri, Barbara Di Camillo, Giacomo Baruzzo, Gabriella De Canal, Alessandra Iurlo, Enrica Morra, Roberto Cairoli

**Affiliations:** 1 Division of Hematology, ASST Grande Ospedale Metropolitano Niguarda, Milano, Italy; 2 Department of Information Engineering, University of Padova, Padova, Italy; 3 Department of Hematology, ASST Spedali Civili, Brescia, Italy; 4 Internal Medicine-Haematology, Desio Hospital, Desio, Italy; 5 Hematology Unit, Fondazione IRCCS Policlinico San Matteo, Pavia, Italy; 6 Division of Hematology, Department of Internal Medicine, Valduce Hospital, Como, Italy; 7 Hematology Division, San Gerardo Hospital, Monza, Italy; 8 Hematology Division, Foundation IRCCS Ca’ Granda Ospedale Maggiore Policlinico, Milano, Italy; 9 Hematology and Bone Marrow Transplantation Unit, San Raffaele Scientific Institute, Milano, Italy; 10 Hematology and Transfusion Medicine, Sacco Hospital, Milano, Italy; 11 Division of Hematology–Fondazione IRCCS Istituto Nazionale dei Tumori, Milano, Italy; 12 ASST Valle Olona Ospedale di Circolo, Busto Arsizio, Italy; 13 ASST Valle Olona Sant’Antonio Abate, Gallarate, Italy; 14 Division of Hematology, Hospital of Cremona, Cremona, Italy; 15 ASST Lecco, Lecco, Italy; 16 Pathology Department, Cytogenetics, ASST Grande Ospedale Metropolitano Niguarda, Milano, Italy; 17 Executive Committee, Rete Ematologia Lombarda, Italy; European Institute of Oncology, ITALY

## Abstract

Chronic myeloid leukemia (CML) is characterized by the constitutive tyrosine kinase activity of the oncoprotein BCR-ABL1 in myeloid progenitor cells that activates multiple signal transduction pathways leading to the leukemic phenotype. The tyrosine-kinase inhibitor (TKI) nilotinib inhibits the tyrosine kinase activity of BCR-ABL1 in CML patients. Despite the success of nilotinib treatment in patients with chronic-phase (CP) CML, a population of Philadelphia-positive (Ph+) quiescent stem cells escapes the drug activity and can lead to drug resistance. The molecular mechanism by which these quiescent cells remain insensitive is poorly understood. The aim of this study was to compare the gene expression profiling (GEP) of bone marrow (BM) CD34+/lin- cells from CP-CML patients at diagnosis and after 12 months of nilotinib treatment by microarray, in order to identify gene expression changes and the dysregulation of pathways due to nilotinib action. We selected BM CD34+/lin- cells from 78 CP-CML patients at diagnosis and after 12 months of first-line nilotinib therapy and microarray analysis was performed. GEP bioinformatic analyses identified 2,959 differently expressed probes and functional clustering determined some significantly enriched pathways between diagnosis and 12 months of nilotinib treatment. Among these pathways, we observed the under expression of 26 genes encoding proteins belonging to the cell cycle after 12 months of nilotinib treatment which led to the up-regulation of chromosome replication, cell proliferation, DNA replication, and DNA damage checkpoint at diagnosis. We demonstrated the under expression of the ATP-binding cassette (ABC) transporters *ABCC4*, *ABCC5*, and *ABCD3* encoding proteins which pumped drugs out of the cells after 12 months of nilotinib. Moreover, GEP data demonstrated the deregulation of genes involved in the JAK-STAT signaling pathway. The down-regulation of *JAK2*, *IL7*, *STAM*, *PIK3CA*, *PTPN11*, *RAF1*, and *SOS1* key genes after 12 months of nilotinib could demonstrate the up-regulation of cell cycle, proliferation and differentiation via MAPK and PI3K-AKT signaling pathways at diagnosis.

## Introduction

CML results from unfaithful repaired DNA damage in a hematopoietic stem cell, but specific features of leukemic stem cells (LSCs) have not yet been fully understood. Several studies demonstrated that LSCs show a strong resistance to therapies in TKI-treated CML patients due to their ability to activate specific signaling biological pathways [1]. Although nilotinib is highly effective in the treatment of CML, multiple clinical trials showed that some patients could become refractory and develop drug resistance [2]. Therapeutic strategies aiming for a cure of CML will require full eradication of Ph+ CML stem cells. Previous studies demonstrated that the aberrant regulation of pathways involved in the self-renewal of stem cells is implicated in cancer [3]. Identifying such pathways and trying to exploit them therapeutically is important to achieve CML-LSC eradication and disease cure [4]. Altered cell cycle checkpoints and a low intracellular concentration of TKIs are among those mechanisms that can lead to drug resistance in CML stem cells [5].

Previous studies demonstrated an increased expression of BCR-ABL1 oncogenic fusion protein-kinase and the deregulation of cell cycle proteins that induced DNA damage in CML cells [6]. These findings highlighted the properties of LSCs which become insensitive and resilient to TKI treatments in the bone marrow niche [7]. In addition, stromal cells play an important role in the survival of LSCs inducing cell cycle arrest and promote cellular quiescence in marginal environments even after TKI therapies [1].

The ABC transporters represent the most abundant transmembrane protein family encoded in the human genome. These membrane proteins transport drugs/substances across the cell membrane by ATP hydrolysis, and their physiological role as a mechanism of defense against xenobiotics has been investigated in CML [8, 9]. An altered regulation of ABC transporter proteins induced multi drug resistance (MDR) in different types of cancer cells [10]. In particular, the over expression of specific ABC transporter proteins can promote drug resistance and the development of malignancy in CML CD34+ population [10]. Indeed, Porro et al, showed that high levels of c-MYC were associated with an increased expression of some members of ABC genes (including *ABCC4*) which were involved in drug resistance in promyeloid leukemia cells [11].

The MDR phenotype may arise not only through the efflux of ABC transporters, but also through several other mechanisms such as pathways involved in the cell growth and survival of LSCs.

In order to identify pathways which contribute to the LSCs survival, several investigations have identified *JAK2* as a putative target for CML. Hematopoietic growth factors (HGFs) bind to specific cell surface receptors in the JAK2-STAT5 cell signaling pathway. Following the HGFs binding, STAT5 is phosphorilated by JAK2 protein within the nucleus. JAK2-STAT5 signaling is involved in the signaling network downstream of BCR-ABL1, playing a crucial role in the leukemogenesis in CML cells [12]. Recently, the existence of a JAK2/BCR-ABL1 protein complex, which helps to stabilize BCR/ABL1 kinase activity, has been demonstrated [13]. Gallipoli et al. concluded that the JAK2/STAT5 signaling pathway is an important therapeutic target in CML stem/progenitor cells, and that JAK2/STAT5 inhibition by nilotinib and ruxolitinib might contribute to obtain disease eradication [12]. Clinical studies combining ruxolinib and TKIs in CML are ongoing in an attempt to eliminate the leukemic stem cell population (EudraCT: NCT01702064).

Gene expression profiling studies have been performed to identify biomarkers predictive of TKI failure [14–16]. In particular, analyses on CML CD34+ cells have revealed that some pathways were consistently deregulated in TKI non-responding patients [1].

The PhilosoPhi34 (EudraCT: 2012-005062-34) study aimed to verify the clearance of BM CD34+/lin- Ph+ cells in CML patients after 3, 6 and 12 months of nilotinib treatment. We investigated the transcriptome profiles and the consequent deregulation of genes and pathways in CD34+/lin- cells from 78 CP-CML patients at diagnosis vs. 12 months of nilotinib treatment by microarray analysis. We determined the deregulation of the cell cycle, the membrane drug-transporters and the JAK-STAT signaling pathway to provide new insight into the action of nilotinib in CP-CML patients.

## Materials and methods

### Patients

The PhilosoPhi34 study, which included 15 centers in Italy, collected samples from consenting patients on behalf of the Rete Ematologica Lombarda (REL). The participants provided their written consent to participate in this study. The study was approved by the Ethics Committee ASST Grande Ospedale Metropolitano Niguarda (Milan, Italy) and the following local Ethics Committees of the participants centers (Lombardia, Italy): EC ASST Spedali Civili Brescia, EC Desio Hospital, EC IRCCS Policlinico San Matteo (Pavia), EC Valduce Hospital (Como), EC Monza Brianza, EC IRCCS Ca’ Granda Ospedale Maggiore Policlinico (Milan), EC San Raffaele Scientific Institute (Milan), EC Sacco Hospital (Milan), EC IRCCS Istituto Nazionale dei Tumori (Milan), EC Valle Olona Ospedale di Circolo (Busto Arsizio), EC ASST Valle Olona Sant’Antonio Abate (Gallarate), EC Hospital of Cremona, and EC ASST Lecco. In this study, we enrolled 87 CP-CML patients [17]. Patients received first-line therapy with nilotinib 300 mg BID.

### Isolation of BM CD34+/lin- cells using immunomagnetic beads

We collected BM samples from 87 patients at diagnosis. In addition, we collected BM samples after 3, 6 and 12 months of nilotinib therapy [17]. 80/87 patients were examined after 12 months of nilotinib. Among these 80 patients, only one relapsed at 12 months. Mononuclear cells (MNCs) from the bone marrow (BM) blood samples (range, 1–25 ml) of 80 CML patients were isolated using Ficoll density gradient centrifugation at 800 rpm for 20 minutes. Immediately afterwards, we selected BM CD34+/lin- cells using Diamond CD34 Isolation kit and autoMACs Pro separator (Miltenyi Biotec, Bologna, Italy) according to the manufacturer’s instructions (Miltenyi Biotec). Briefly, we labeled BM MNCs with a mix of biotin-conjugated antibodies against lineage-specific antigens. Immediately afterwards, these cells were labeled with Anti-Biotin Microbeads. We selected the lineage-negative stem and progenitor cells by the depletion of the magnetically labeled cells. BM CD34+/lin- cells were obtained from the lineage-negative stem and progenitor cells using CD34 Microbeads (Miltenyi Biotec). The purity of isolated BM CD34+/lin- cells was detected by flow cytometry.

The methods were described in http://dx.doi.org/10.17504/protocols.io.yncfvaw, and showed in our previous study [18].

### FISH

Standard FISH tests were performed on isolated BM CD34+/lin- cells for 87 patients at diagnosis and for 80/87 patients after 3, 6 and 12 months of nilotinib treatment. For each patient, a small sample of selected CD34+/lin- cells (containing at least 10^3^ cells fixed in Carnoy’s solution) was analyzed by FISH using standard method [18]. Samples were co-hybridized to XL BCR/ABL1 plus Translocation/Dual Fusion Probe (MetaSystems, Milan, Italy) on ThermoBrite Statspin Model (Leica Biosystems, US). FISH analyses were performed using fluorescence microscope Axioskop 2 (Carl Zeiss Microimaging GmbH, Göttingen, Germany), equipped with a UV 100-W lamp (Osram, Augsburg, Germany), ProgRes MF CCD camera (Jenoptik AG, Jena, Germany), and ISIS System Software (MetaSystems Hard & Software, Althlussheim, Germany).

#### Fine modulo

At least, 200 interphase nuclei were counted from each suitable specimen (optimum: 300 nuclei). Each available interphase nucleus was read even in sub-optimal specimens. FISH analyses were performed as described by Trojani et al [18], and in http://dx.doi.org/10.17504/protocols.io.yncfvaw.

### Cell cryopreservation and RNA extraction

Selected BM CD34+/lin- cells of 80 CP-CML patients were resuspended in 50 μl of RNAlater (Thermo Fisher Scientific, Milano, Italy) and stored at -20°C until RNA extraction was performed as previously described [18].

Total RNA was isolated from the BM CD34+/lin- cells stored in RNAlater using MagMAX 96 Total RNA Isolation Kit (Thermo Fisher Scientific) [18], according to the manufacturer’s instructions. The quality and the yield of the extracted RNA were measured using Nanodrop (Thermo Fisher Scientific) (see http://dx.doi.org/10.17504/protocols.io.yncfvaw).

### GEP experiments

Microarray experiments were performed on the BM CD34+/lin- cells of 80 CP-CML patients at diagnosis as well as those who had undergone 12 months of nilotinib treatment. We prepared cDNA starting from the previously extracted RNA (50 ng) using Ovation Pico WTA System V2 kit (NuGEN) and Encore Biotin Module Kit (NuGEN) following the manufacturer’s instructions.

cDNA was hybridized to Affymetrix HTA 2.0 using the Gene Chip platform (Affymetrix, Santa Clara, Ca, USA) and signals were scanned by Affymetrix Gene Chip Scanner 3000 according to the manufacturer’s instructions as described in http://dx.doi.org/10.17504/protocols.io.yncfvaw, and in our previous manuscript [18].

### Bioinformatic analyses of GEP data

The preprocessing of microarray raw data was performed using R software version 3.4.2 [19]. The Affymetrix HTA 2.0 probes were initially summarized into probe sets specific for a given gene using function *RMA* [20] of R package *oligo* [21], downloaded from Bioconductor repository version 3.4. Principal component analysis (PCA) has been performed using *prcomp* function of package *stats* version 3.4.2 [19]. MvA plots were generated using custom scripts. MvA plots show the relationship among the average log intensity of the gene expression (A value) and the log of intensity ratio (M value) between two samples. PCA and MvA plots were examined before and after microarray preprocessing as a quality checking procedure. PCA plots revealed the presence of batch effects due to the different protocols used for performing RNA extraction and GEP experiments. Batch effects have been corrected using function *ComBat* [22] of R package *sva* [23]. MvA plots showed the presence of bias in the distribution of intensities among samples, then data was normalized using function *normalize*.*quantiles* of R package *preprocessCore* [24].

The differential expression analysis was performed on the samples at 12 months vs. diagnosis using the two-classes SAM test [25], implemented in the homonym function in R package *samr* [25]. Benjamini-Hochberg procedure was applied to control the False Discovery Rate (FDR) and a cut-off value of 0.05 was applied to select for significant differential expression [26].

Functional clustering was performed on significant differentially expressed genes using online tool DAVID (https://david.ncifcrf.gov/) [27, 28], to classify them into functional groups based on their annotation term co-occurrence. For this analysis, 1,723 protein coding genes which have a unique EntrezID in the “Affymetrix NetAffx” annotation were used (HTA 2.0 Transcript Cluster Annotations, Release 36, 7/6/16). Groups that resulted significantly enriched were selected based on FDR value below 0.05 [29], (see http://dx.doi.org/10.17504/protocols.io.yncfvaw).

## Results

### FISH

At diagnosis, FISH analysis detected BM CD34+/lin- Ph+ cells in all 87 CP-CML patients. At 12 months, we could analyze 80/87 patients [17]. 79/80 patients were evaluable because they achieved at least a complete cytogenetic response whereas 1/80 patient relapsed at 12 months. No Ph+ nuclei were detected in 79/79 patients [17].

### Purity of selected cells, quality and yield of total RNA

The purity of BM CD34+/lin- cells was > 97% as determined by flow cytometry (S1 Appendix). The purity of the extracted RNA was in the range of 1.7–1.8, determined by absorbance ratios of A(260)/A(280) using a NanoDrop Spectrophotometer (Thermo Fisher Scientific). The total RNA concentration isolated from 100,000 BM CD34+/lin- cells was about 300 ng.

### Preprocessing of HTA 2.0 arrays of BM CD34+/lin- cells of CP-CML patients at diagnosis and after 12 months of nilotinib treatment

We performed the preprocessing and correction for batch effects for samples of 80 patients at diagnosis and after 12 months of nilotinib treatment. We conducted the analyses on 78 subjects. Due to experimental issues, two patients were not considered for differential expression analysis, as the microarray CEL files of the 12 months samples were corrupted and missed probe intensities for most of the probes. After correction for batch effects and normalization, no more batch effects or residual systematic differences were observed in all the 156 arrays.

### Identification of genes and pathways deregulated between BM CD34+/lin- cells of CP-CML patients at diagnosis vs. 12 months of nilotinib treatment

The differential expression analysis detected 2,959 probes (corresponding to 2,726 unique genes and 1,740 unique gene symbols) differently expressed (DE) between 78 patients at diagnosis compared to 12 months of nilotinib treatment (S1 Table). Among the unique genes, 1,868 genes were annotated as “protein coding” and 858 as “non-coding” in the “Affymetrix NetAffx” annotation (HTA 2.0 Transcript Cluster Annotations, Release 36, 7/6/16). Most of non-coding DE genes (364 genes) consisted of long non-coding RNAs, while the remaining genes were annotated as snoRNAs, miRNAs, piRNAs, miscRNAs, tRNAs and rRNAs.

The functional clustering analysis revealed interesting functional groups of genes, involved in cell cycle, ATP-binding, and JAK-STAT pathway (Table 1 and S2 Table).

**Table 1:** Genes with significant differential expression in BM CD34+/lin- cells from 78 CP-CML patients at diagnosis vs. 12 months of nilotinib treatment.

Gene Symbol^a^	Fold Change^b^	Adjusted p-value^c^	KEGG Pathway^d^
ANAPC1	1.23	0.01	CELL CYCLE AND MITOSIS
ANAPC4	1.20	0.01	CELL CYCLE AND MITOSIS
ANAPC7	1.16	0.04	CELL CYCLE AND MITOSIS
ATM	1,24	0,01	CELL CYCLE AND MITOSIS
BUB1	1.16	0.04	CELL CYCLE AND MITOSIS
BUB3	1.23	0.02	CELL CYCLE AND MITOSIS
CCNA2	1.29	0.01	CELL CYCLE AND MITOSIS
CCNB1	1.26	0.02	CELL CYCLE AND MITOSIS
CDC27	1.27	0.01	CELL CYCLE AND MITOSIS
CDC6	1.21	0.02	CELL CYCLE AND MITOSIS
CDK7	1.17	0.04	CELL CYCLE AND MITOSIS
DBF4	1.19	0.02	CELL CYCLE AND MITOSIS
HDAC2	1.33	0.02	CELL CYCLE AND MITOSIS
MAD2L1	1.27	0.01	CELL CYCLE AND MITOSIS
MCM3	1.19	0.02	CELL CYCLE AND MITOSIS
MCM6	1.25	0.03	CELL CYCLE AND MITOSIS
MDM2	1.26	0.02	CELL CYCLE AND MITOSIS
ORC2	1.31	0.01	CELL CYCLE AND MITOSIS
ORC4	1.19	0.03	CELL CYCLE AND MITOSIS
ORC5	1.17	0.02	CELL CYCLE AND MITOSIS
PRKDC	1.27	0.01	CELL CYCLE AND MITOSIS
SMC3	1.26	0.02	CELL CYCLE AND MITOSIS
TTK	1.24	0.02	CELL CYCLE AND MITOSIS
WEE1	1.31	0.01	CELL CYCLE AND MITOSIS
YWHAE	1.38	0.01	CELL CYCLE AND MITOSIS
YWHAZ	1.36	0.05	CELL CYCLE AND MITOSIS
ABCD3	1.19	0.03	ABC TRANSPORTERS
ABCC5	1.09	0.04	ABC TRANSPORTERS
ABCC4	1.21	0.01	ABC TRANSPORTERS
IL22RA1	-1.13	0.02	JAK-STAT
SOS1	1.32	0.01	JAK-STAT
PIK3CA	1.24	0.01	JAK-STAT
RAF1	1.11	0.03	JAK-STAT
IL7	1.16	0.03	JAK-STAT
JAK2	1.21	0.02	JAK-STAT
STAM	1.29	0.01	JAK-STAT
PTPN11	1.18	0.03	JAK-STAT

^a^ Official gene symbols.

^b^ Fold changes of gene expression (12 months vs. diagnosis).

^c^ P-value adjusted according to Benjamini-Hochberg false discovery rate.

^d^ KEGG pathway name.

### Up-regulation of 26 genes of the Cell Cycle (G_1_, S, G_2_ and M phases), DNA damage and repair at diagnosis

**Fig 1:**
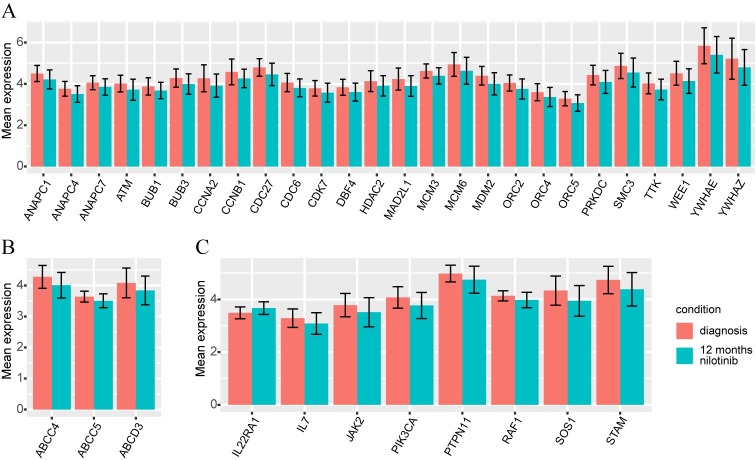
(A) Box plot of expression of genes of the Cell Cycle and Mitosis pathway. Twenty-six genes were significantly differentially expressed in BM CD34+/lin- cells from CP-CML patients at diagnosis vs. 12 months of nilotinib treatment. (B) Box plot of expression of genes of the ATP-binding cassette (ABC) pathway. The comparison between BM CD34+/lin- cells of CP-CML patients at diagnosis and 12 months of nilotinib treatment showed 3 genes significantly differentially expressed. (C) Box plot of expression of genes of the JAK-STAT pathway. Eight genes demonstrated a significant differential expression in BM CD34+/lin- cells from CP-CML patients at diagnosis vs. 12 months of nilotinib treatment.

Functional enrichment analysis demonstrated that 26/124 genes encoding proteins that belong to the cell cycle pathway were significantly over expressed at diagnosis compared to 12 months of nilotinib (Tables 1 and 2, Fig 1A). *ORC5*, *ORC2*, *ORC4* (Origin Recognition Complex), *MCM3*, *MCM6* (Mini-Chromosome Maintenance complex) and *HDAC2* encoding proteins that belong to G_1_ phase of the cell cycle (Cell cycle control of Chromosome replication), were up-regulated at diagnosis. We demonstrated that *CCNA2*, *CDK7*, *CDC6*, *DBF4*, *ORC5*, *ORC2*, *ORC4*, *MCM3*, and *MCM6* (S phase of the cell cycle) were over expressed at diagnosis. GEP results showed that *CCNA2*, *CCNB1*, *WEE1*, *PRKDC*, *ATM*, *MDM2* (G2 phase of the cell cycle) as well as *TTK*, *MAD2L1*, *BUB3*, *BUB1*, *ANAPC1*, *ANAPC4*, *ANAPC7*, *CDC27*, *SMC3*, *YWHAE*, and *YWHAZ* (M phase of the cell cycle) were over expressed at diagnosis compared to 12 months of nilotinib.

**Table 2:** Genes of the Cell cycle and Mitosis pathway with significant differential expression in BM CD34+/lin- cells from 78 CP-CML patients at diagnosis vs. 12 months of nilotinib treatment.

Gene Symbol^a^	Fold Change^b^	Adjusted p-value^c^
ANAPC1	1.23	0.01
ANAPC4	1.20	0.01
ANAPC7	1.16	0.04
ATM	1,24	0,01
BUB1	1.16	0.04
BUB3	1.23	0.02
CCNA2	1.29	0.01
CCNB1	1.26	0.02
CDC27	1.27	0.01
CDC6	1.21	0.02
CDK7	1.17	0.04
DBF4	1.19	0.02
HDAC2	1.33	0.02
MAD2L1	1.27	0.01
MCM3	1.19	0.02
MCM6	1.25	0.03
MDM2	1.26	0.02
ORC2	1.31	0.01
ORC4	1.19	0.03
ORC5	1.17	0.02
PRKDC	1.27	0.01
SMC3	1.26	0.02
TTK	1.24	0.02
WEE1	1.31	0.01
YWHAE	1.38	0.01
YWHAZ	1.36	0.05

^a^ Official gene symbols.

^b^ Fold changes of gene expression (12 months vs. diagnosis).

^c^ P-value adjusted according to Benjamini-Hochberg false discovery rate.

### Over expression of ATP-binding ABC transporters genes in CD34+/lin- cells at diagnosis

GEP data demonstrated that *ABCC4*, *ABCC5* and *ABCD3* genes were significantly up-regulated at diagnosis (Tables 1 and 3, Fig 1B). We previously demonstrated the over expression of *ABCC5* at diagnosis vs. 12 months of nilotinib treatment in 30 CP-CML patients [18].

**Table 3:** Genes of the ATP-binding cassette (ABC) pathway with significant differential expression in BM CD34+/lin- cells from 78 CP-CML patients at diagnosis vs. 12 months of nilotinib treatment.

Gene Symbol^a^	Fold Change^b^	Adjusted p-value^c^
ABCD3	1.19	0.03
ABCC5	1.09	0.04
ABCC4	1.21	0.01

^a^ Official gene symbols.

^b^ Fold changes of gene expression (12 months vs. diagnosis).

^c^ P-value adjusted according to Benjamini-Hochberg false discovery rate.

### Activation of JAK-STAT signaling pathway at diagnosis vs. 12 months of nilotinib

We analyzed JAK-STAT signaling pathway that is made up of 155 genes (Kegg Pathway Database). This pathway was deregulated in CD34+/lin- cells at diagnosis vs. 12 months of nilotinib treatment. *SOS1*, *PIK3CA*, *RAF1*, *IL7*, *JAK2*, *STAM*, and *PTPN11* were up-regulated whereas *IL22RA* was down-regulated at diagnosis (Tables 1 and 4, Fig 1C).

**Table 4:** Genes of the JAK-STAT pathway with significant differential expression in BM CD34+/lin- cells from 78 CP-CML patients at diagnosis vs. 12 months of nilotinib treatment.

Gene Symbol^a^	Fold Change^b^	Adjusted p-value^c^
IL22RA1	-1.13	0.02
SOS1	1.32	0.01
PIK3CA	1.24	0.01
RAF1	1.11	0.03
IL7	1.16	0.03
JAK2	1.21	0.02
STAM	1.29	0.01
PTPN11	1.18	0.03

^a^ Official gene symbols.

^b^ Fold changes of gene expression (12 months vs. diagnosis).

^c^ P-value adjusted according to Benjamini-Hochberg false discovery rate.

## Discussion

The resistance to TKIs remains one of the major causes of treatment failure and patient death in CML [30]. A better understanding of the molecular biology of LSCs is crucial to develop more effective treatments for advanced CML and prevent drug resistance [1].

To the best of our knowledge, we hereby report for the first time the results of a wide transcriptome analysis of BM CD34+/lin- cells of 78 CP-CML patients at diagnosis vs. 12 months of nilotinib treatment. We found 2,959 probes differently expressed at diagnosis compared to 12 months of nilotinib treatment. In particular, we focused on genes which are over expressed at diagnosis and which play a crucial role in the cell cycle, ATP-binding ABC transporters and JAK-STAT signaling pathway (Fig 1).

Gene expression and proteomic profile studies of CML LSCs drew attention to specific gene pathways that could represent both prognostic indicators as well as new targets for therapy that might eventually overcome resistance to the BCR-ABL TKIs [31, 32].

The alteration of different signaling pathways such as cell cycle, JAK-STAT, and the deregulation of ABC drug efflux transporters can promote the development of growth and survival of CML progenitor and stem cells [1]. Some authors showed that several genes encoding proteins involved in the cell cycle and chromosome segregation were up-regulated in CML LSCs [1]. We demonstrated that 26 genes representing phases of the cell cycle (G_1_, S, G_2_ and M), were over expressed at diagnosis compared to 12 months of nilotinib treatment in 78 CP-CML patients (Tables 1 and 2). The integrity of signaling pathways involved in cell cycle arrest, chromatin remodeling and DNA repair are critical to maintain the fidelity of replicated DNA. Mancini et al, demonstrated that normal cells repaired damaged DNA during G_1_ arrest whereas leukemic cells often had a deficient G_1_/S checkpoint and this depended on a functional G_2_/M checkpoint for DNA repair (Mancini M. et al. Blood. 2017; Abs. Suppl1 130:1588).

Among the 26 deregulated genes, we found that *ORC5*, *ORC2*, *ORC4*, *MCM3*, *MCM6*, and *HDAC2* controlled G_1_ phase as well chromosome replication. The up-regulation of these genes was associated with the initiation of DNA replication [33]. Notably, some studies demonstrated that HDAC inhibitors treatment represented an effective strategy to target LSCs in CP-CML patients receiving tyrosine kinase inhibitors [34, 35].

Our GEP results demonstrated that genes encoding proteins involved in the S phase of cell cycle (*CCNA2*, *CDK7*, *CDC6*, *DFB4*, *MCM3*, and *MCM6*) were down-regulated after 12 months of nilotinib. Previous studies showed that these genes might promote the cell proliferation and DNA replication in CML CD34+/lin- cells at diagnosis [15, 36, 37].

We showed that *CCNA2*, *CCNB1*, *WEE1*, *PRKDC*, *ATM* and *MDM2* (G_2_ phase) were down-regulated after 12 months of nilotinib. Notably, the study by Reynaud et al, demonstrated the over expression of *CCNA2* and *CCNB1* in CML-LSCs of transgenic mice [38].

Our study demonstrated that *TTK*, *MAD2L1*, *BUB3*, *BUB1*, *ANAPC1*, *ANAPC4*, *ANAPC7*, *CDC27*, *SMC3*, *YWHAE*, and *YWHAZ* (mitosis) were over expressed at diagnosis. In particular, *TTK* and *MAD2L1* might increase cell proliferation in CML CD34+/lin- cells at diagnosis, and some researchers demonstrated that they were over expressed in CML leukemic stem cells compared to the same cell counterpart from normal subjects [15]. Moreover, previous studies showed the over expression of the mitotic checkpoint genes *BUB1* and *BUB2* in several solid tumors [39–41].

In conclusion, we can reasonably speculate that all the 26 genes over expressed at diagnosis led to the up-regulation of the cell cycle in CML CD34+/lin- cells at diagnosis increasing their survival with respect to 12 months of nilotinib treatment.

Our results showed that *ABCC5*, *ABCC4* and *ABCD3* were significantly under expressed in CP-CML patients after 12 months of nilotinib treatment compared to diagnosis [18]. Previous studies demonstrated that drug transporters, particularly ATP-binding cassette (ABC) transporters, played a critical role in the intracellular levels of TKI and primary resistance [10]. Indeed, 48 genes represent the ABC transporters family (Kegg Pathway Database), and the up-regulation of some of them can lead to MDR by promoting the efflux of drugs out of the cells [9, 10, 11, 42]. Recent studies have investigated *ABCC4* and *ABCC5* to clarify the clinical significance of their altered function and expression in MDR. In particular, Chen et al, demonstrated that proteins encoded by *ABCC4* and *ABCC5* were expressed at low levels in all normal tissues [42]. Wang et al, demonstrated that both *ABCC4* and *ABCC5* regulated the efflux of purine analogues. In order to overcome the drug resistance, recent *in vitro* studies demonstrated that TKIs such as nilotinib and imatinib were able to inhibit the efflux actions of ABC transporter proteins [43–46].

Several studies on CML demonstrated JAK-STAT signaling pathway as a potential survival mechanism of CML LSCs [4]. Recently, researchers focused on the function of the intracellular *JAK2* in the survival and proliferation of CML LSCs and its putative role as a therapeutic target in CML [12]. The combination of JAK2 inhibitors with TKI showed to be effective against CML cell lines and primary cells. However, further work is still required to assess the effectiveness, toxicity and specificity of inhibitors [31, 47–49]. Some studies are ongoing to identify other regulators of the JAK-STAT pathway and to design innovative therapeutic strategies. Our GEP data demonstrated an average up-regulation of 7 genes (*JAK2*, *SOS1*, *PIK3CA*, *RAF1*, *IL7*, *STAM*, and *PTPN11*) encoding proteins of JAK-STAT signaling pathway at diagnosis.

In addition, the JAK-STAT pathway plays a major role in the transfer of signals from cell-membrane receptors to the nucleus [50]. The interaction between the surface receptors and the cytokines activates *JAK2* and the cascade of genes which lead to the proliferation, differentiation, cell cycle and survival of LSCs. Our study identified the dysregulation of MAPK and PI3K signaling pathways due to the over expression of *PTPN11*, *SOS1*, *RAF1* and *PIK3C*, respectively [51–53]. Moreover, *JAK2* could promote the phosphorilation of PIK3CA via PI3K-AKT signaling pathway [53] that might be responsible to TKI resistance in Ph+ cell lines [54].

In summary, we identified gene expression changes in BM CD34+/lin- cells of a cohort of 78 CP-CML patients after 12 months of nilotinib therapy compared to diagnosis. The dysregulation of cell cycle and DNA repair, ABC transporters, and JAK-STAT signaling pathway after treatment with nilotinib are interesting, since previous studies highlighted the role of these pathways in CML.

We determined that the BM CD34+/lin- cells at diagnosis were all Ph-positive whereas the same cells after 12 months of nilotinib were Ph-negative by FISH analyses. We could suppose that BM CD34+/lin- cells of patients after 12 months of nilotinib were normal because of the cytogenetic results. To clarify this point, we will compare GEP of BM CD34+/lin- cells after 12 months of nilotinib with respect to the normal cell counterparts of healthy donors.

The potential suitability of the genes highlighted in our study as biomarkers in CML requires, however, further investigation to address their clinical relevance. We strongly believe that the identification of dysregulated signaling pathways in progenitor and stem cells in CML patients can significantly alter the presentation of the disease and its progression, and therefore might suggest the design of new therapeutic strategies in CML. Furthermore, the identification of pathways that might represent new drug-targets for elimination of LSCs, could improve the outcomes of CML patients.

## Supporting information

S1 Appendix A representative FACS plot of the purity of BM CD34+/lin- cells determined by flow cytometry. (PPTX)

S1 Table Results of the differential expression analysis on 78 CML patients at diagnosis vs. 12 months of treatment with nilotinib. (XLSX)

S2 Table Results of the functional clustering analysis performed with DAVID tool on differentially expressed genes. (XLSX)
