# Supplementary material for: Nilotinib interferes with cell cycle, ABC transporters and JAK-STAT signaling pathway in CD34+/lin- cells of patients with chronic phase chronic myeloid leukemia after 12 months of treatment
Source: PLoS One. 2019 Jul 18;14(7):e0218444. doi: 10.1371/journal.pone.0218444 (PMC6638825; doi:10.1371/journal.pone.0218444)
Supplement: S1 Appendix — (PPTX) [file pone.0218444.s001.pptx]

## Slide 1
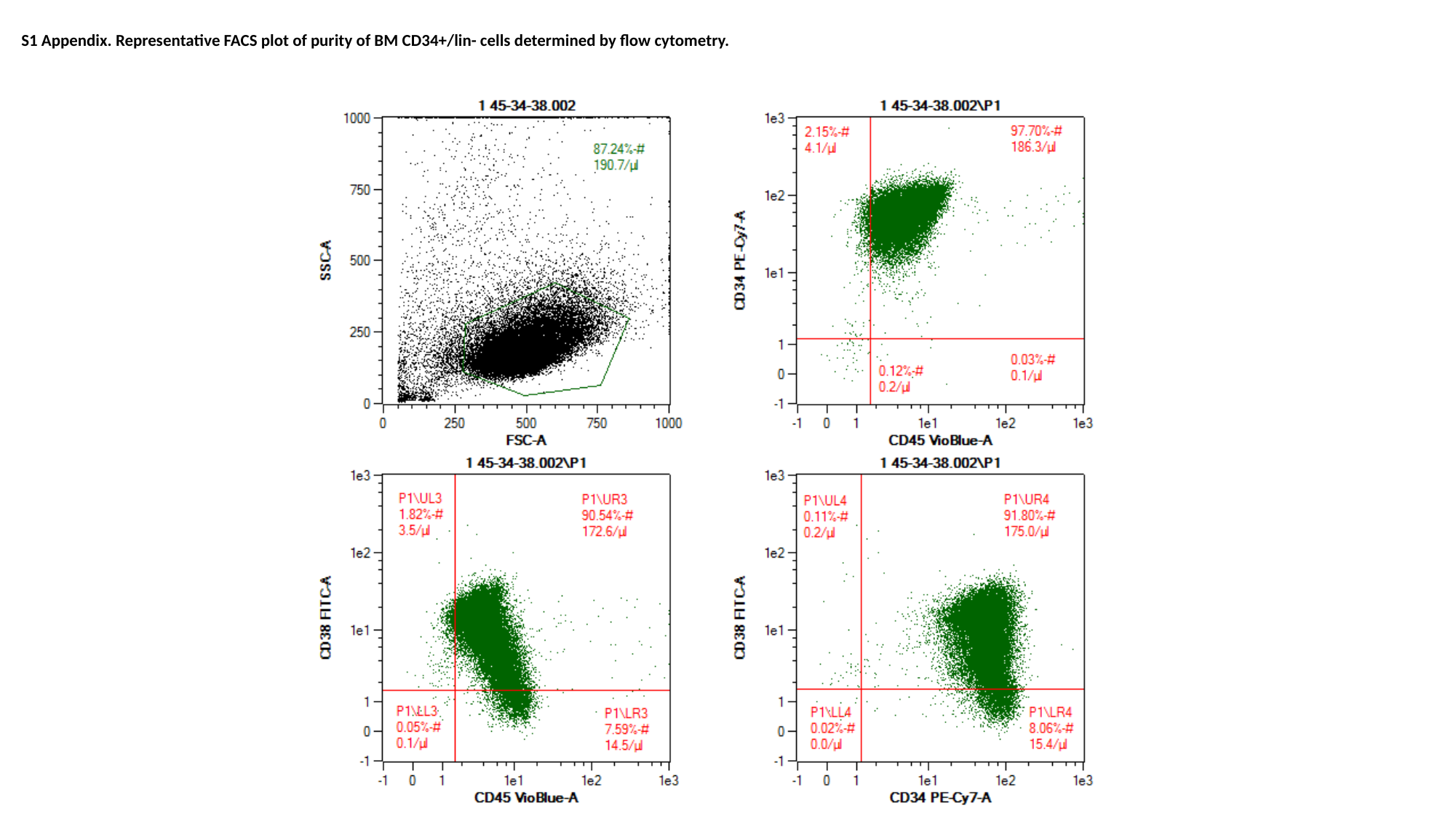

S1 Appendix. Representative FACS plot of purity of BM CD34+/lin- cells determined by flow cytometry.
